# The incidence of sub-optimal sedation in the ICU: a systematic review

**DOI:** 10.1186/cc8212

**Published:** 2009-12-16

**Authors:** Daniel L Jackson, Clare W Proudfoot, Kimberley F Cann, Tim S Walsh

**Affiliations:** 1 GE Healthcare, Pollards Wood, Nightingales Lane, Chalfont St. Giles, Bucks, HP8 4SP, UK; 2 Heron Evidence Development Ltd, Building 210A, Butterfield Technology and Business Park, Luton, LU2 8DL, UK; 3 Royal Infirmary of Edinburgh, 51 Little France Crescent, Old Dalkeith Road, Edinburgh, EH16 2SA, UK

## Abstract

**Introduction:**

Patients in intensive care units (ICUs) are generally sedated for prolonged periods. Over-sedation and under-sedation both have negative effects on patient safety and resource use. We conducted a systematic review of the literature in order to establish the incidence of sub-optimal sedation (both over- and under-sedation) in ICUs.

**Methods:**

We searched Medline, Embase and CINAHL (Cumulative Index to Nursing and Allied Health Literature) online literature databases from 1988 to 15 May 2008 and hand-searched conferences. English-language studies set in the ICU, in sedated adult humans on mechanical ventilation, which reported the incidence of sub-optimal sedation, were included. All abstracts were reviewed twice by two independent reviewers, with all conflicts resolved by a third reviewer, to check that they met the review inclusion criteria. Full papers of all included studies were retrieved and were again reviewed twice against inclusion criteria. Data were doubly extracted. Study aims, design, population, comparisons made, and data on the incidence of sub-optimal, optimal, over-sedation or under-sedation were extracted.

**Results:**

There was considerable variation between included studies in the definition of optimal sedation and in the scale or method used to assess sedation. Across all included studies, a substantial incidence of sub-optimal sedation was reported, with a greater tendency toward over-sedation.

**Conclusions:**

Our review suggests that improvements in the consistent definition and measurement of sedation may improve the quality of care of patients within the ICU.

## Introduction

The majority of mechanically ventilated patients within the intensive care unit (ICU) receive sedative drugs. Sedation is administered to ensure patient comfort, reduce anxiety, and facilitate treatments. Optimising sedation management is recognised as important in improving patient outcomes [1]. Under-sedated patients may become agitated and distressed and are at risk of adverse events such as extubation [2-4], whereas over-sedation can prolong time to recovery [1,5].

Assessment of sedation level is carried out mainly by nurses or critical care physicians by assessing patient responses to simple stimuli. Sedation scales such as the Ramsay scale or the Richmond Agitation-Sedation Scale (RASS) are widely used [6-8]. However, there is no universally accepted standard, and this can make comparison between different studies or ICUs difficult [2]. Furthermore, some of these scales have not been fully validated in ICU patients [4]. Recently, devices such as the bispectral index monitor (BIS), which aim to assess sedation levels more objectively, have been introduced. However, most studies of BIS have been performed in surgical settings, and to date its effectiveness is not fully proven [8-10].

Available guidelines on sedation typically provide limited guidance on optimal sedation monitoring and levels. This is at least partly because optimal sedation levels differ between patients according to their clinical circumstances, and therefore sedation practice is ideally individually tailored to each patient, as recommended by several guidelines [2,11,12]. However, among guidelines that do recommend an optimal level of sedation, there are discrepancies, indicating a lack of consensus on this issue. For example, of a survey of available guidelines, one [13] recommended a sedation level of 2 or 3 on the Ramsay scale, whereas one recommended a goal of RASS -3 for an intubated patient [14] and a second recommended a goal of RASS 0 to -2 [15]. A number of guidelines stress the importance of establishing a set protocol for the sedation of ICU patients [16,17] but do not set out such a protocol in detail, leaving it to individual institutions, and more recent guidelines recognise the benefit of regular (daily) interruption of sedation for eligible patients [11,14,18,19] within sedation protocols.

It is recognised that optimising sedation practice is a recognised quality marker for intensive care treatment, and procedures designed to optimise patient sedation state, such as daily sedation breaks and more frequent monitoring, are key elements of recent quality improvement initiatives. However, despite these recent efforts to improve the quality of sedation practice in the ICU, the epidemiology of sedation, and specifically the prevalence of over- or under-sedation, is unclear. To investigate this further, we carried out a systematic review of the publicly available literature to identify the reported incidence of sub-optimal sedation.

## Materials and methods

### Searching

Medline, Embase and the Cumulative Index to Nursing and Allied Health Literature (CINAHL) databases were searched from 1988 to 15 May 2008 using terms for sedation, ICU, sedation quality management, and sub-optimal sedation. The standard Scottish Intercollegiate Guidance Network (SIGN) filters for randomised controlled trials (RCTs), economic studies and observational studies [20] were combined to capture all study designs relevant to the study question. Full details of the search strategy used are available from the authors on request. Conference proceedings from 2005 through 2008 were hand-searched for relevant studies. All results were uploaded into a bespoke internet SQL (structured query language)-based database.

### Selection criteria

Inclusion of studies was according to a predetermined set of criteria. To be included, studies had to be in adult humans who were sedated and undergoing mechanical ventilation within the ICU and furthermore had to report the incidence of sub-optimal sedation, over- or under-sedation, or of optimal sedation, as defined by the study. Studies that reported the impact of sedation practice on outcomes were also included; these data are reported separately. In addition, short-term studies (including only patients sedated less than 24 hours) were excluded. Only English-language studies were included. To check that they met the review inclusion criteria, all abstracts were reviewed twice by two independent reviewers, with all conflicts resolved by a third reviewer. Full papers of all included studies were retrieved and were again reviewed twice to ensure that they met inclusion criteria. Studies included at this stage were classified as to which aspect of the review question they met, and appropriate data were extracted, summarised and analysed.

### Data extraction

Data were extracted by two reviewers and checked by a third reviewer against the original studies. For all studies, the following data were extracted: country, sponsor, study design, patient population, objective, number of patients in the study, details of comparisons made (such as between different treatment arms or between different sedation monitoring systems), and the proportion of measurements, patients, or time in which patients were judged to be optimally sedated, sub-optimally sedated, over-sedated, or under-sedated.

### Quantitative data synthesis

Due to the wide range of included study types, no studies were suitable for quantitative data synthesis.

## Results

### Systematic review study flow

**Figure 1:**
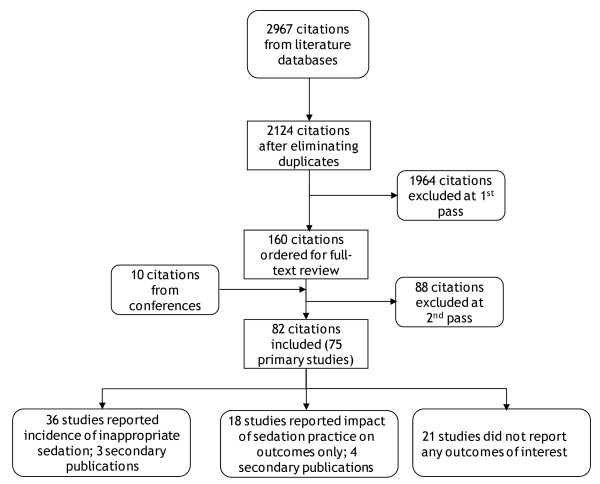
The QUOROM (Quality of Reporting of Meta-Analyses) diagram illustrates the flow of studies through the systematic review.

The flow of studies through the systematic review is documented in the QUOROM (Quality of Reporting of Meta-Analyses) diagram in Figure 1. Seventy-five primary and seven secondary studies met the inclusion criteria. Of these, 18 did not provide any data; either they did not contain data on the outcomes extracted in this review or they did not provide these data in quantitative form. Thirty-six studies reported data on the incidence of sub-optimal sedation. The remainder reported the impact of sedation practice on outcomes; these data are reported separately. Of the included studies, three were cohort studies that specifically investigated the epidemiology of sedation, 23 were studies investigating anaesthetic drugs (of which 19 were RCTs and four were observational studies), six studies compared sedation monitoring devices or scales (of which one was an RCT and the remainder were observational studies), three studies investigated the introduction of sedation guidelines, and one did not fit any of these categories. The majority of studies (20) were published after 2002, indicating the increasing interest in the practice of sedation quality in recent years, in particular following the publication of updated sedation guidelines from the American College of Critical Care Medicine [2,6].

### Definitions of adequate sedation

To assess the incidence of sub-optimal sedation, it is necessary to consider the definition of what constitutes optimal sedation. We used the definition of optimal sedation (and consequently of what constituted sub-optimal sedation) provided by individual studies due to the fact that optimal sedation levels will vary according to study setting (for example, between neurological ICU and medical ICU).

**Figure 2:**
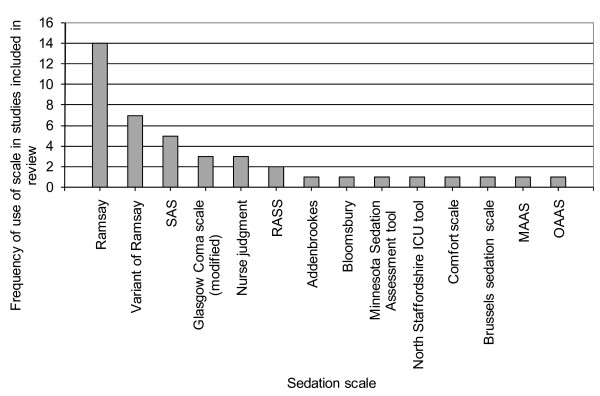
The frequency with which each sedation scale was used in the studies included in our systematic review. ICU, intensive care unit; MAAS, Motor Activity Assessment Scale; OAAS, Observer's Assessment of Alertness/Sedation Scale; RASS, Richmond Agitation Sedation Scale; SAS, Riker Sedation-Agitation Scale.

Across all of the studies, 13 different sedation scales were used to assess sedation quality; additionally, nurse assessment of sedation quality simply as over-sedated, under-sedated, or adequate was used three times (Table 1). The Ramsay scale was the most commonly used scale, in 14 studies, with a variant used in a further 7 studies. This is illustrated in Figure 2.

**Table 1:** Incidence of optimal and sub-optimal sedation in included studies

Study	Study design and comparisons made	Number	Treatment arms (if relevant)	Incidence of sub-optimal sedation	Incidence of over-sedation	Incidence of under-sedation	Incidence of optimal sedation	Sedation scale/monitoring system used	Definition of optimal sedation
Weinert, *et al*., 2007 [44]	Cohort study	274			326 (2.6%) of 12,414 assessments. 111 patients (40%) had ≥ 1 rating of over-sedation. Patients were unarousable/minimally arousable 32% of the time.	1,731 (13.9%) of 12,414 assessments. 211 (76.2%) had ≥ 1 rating of under-sedation.	10,357 (83%) of 12,414	Minnesota Sedation Assessment Tool -- nurse assessment	Arousal level 3-5 (of 6-point scale)
Martin, *et al*., 2006 [30]	Cohort study	305 (from 220 ICUs)			42.6% of 49 patients sedated 24-72 hours, 39.5% of 157 patients sedated >72 hours, and 43.9% of 57 patients under weaning had significantly deeper sedation than desired level	5.2% of 157 patients sedated >72 hours and 3.5% of 57 patients under weaning had significantly lower sedation than desired level	In patients sedated >72 hours, the desired Ramsay score was 0-4 in 44% of cases -- this was achieved in 28%; in 55% of patients, the desired value was 4-5, which was achieved in 68%; in 1% of patients, the desired score was 6, which was achieved in 6%.	Ramsay scale	Individual to each patient
Payen, *et al*., 2007 [43]	Cohort study	1,381			258 (57%) of 451 patients on sedation day 2; 169 (48%) of 355 patients on day 4; 109 (41%) of 266 patients on day 6			Multiple: most commonly Ramsay, RASS, Sedation-Agitation scale	Over-sedation defined as Ramsay 5-6, RASS -5 or --4, Sedation-Agitation scale 1-2
Sandiumenge, *et al*., 2000 [36]	RCT/observational study of sedative drugs	63	Midazolam	19 (7%) of 266 hours			247 (93%) of 266 hours	Modified Ramsay scale	Equivalent of Ramsay 5-6 (for deep sedation)
			2% propofol	14 (9%) of 156 hours			142 (91%) of 156 hours		
Carrasco, *et al*., 1993 [26]	RCT (with economic study) of sedative drugs	88	Midazolam	18% of time (hours)			82% of time (hours)	Ramsay scale; Glasgow coma scale (modified by Cook and Palma)	Ramsay scale 2-5, Glasgow coma scale 8-13
			Propofol	7% of time (hours)			93% of time (hours)		
McCollam, *et al*., 1999 [23]	RCT of sedative drugs	30	Lorazepam	32% of assessments	14% of assessments	18% of assessments	68% of assessments	Ramsay scale	Ramsay scale 2-4
			Midazolam	21% of assessments	6% of assessments	16% of assessments	79% of assessments		
			Propofol	38% of assessments	7% of assessments	31% of assessments	62% of assessments		
Chinachoti, *et al*., 2002 [40]	RCT of sedative drugs	152	Remifentanil	28% of patients; 17.3% of time (hours)	13% of time (hours)	4% of time (hours)	78% of patients (without midazolam), 83% of time (hours) (maintenance phase)	SAS	SAS 4 with no or mild pain
			Morphine	27% of patients; 16% of time (hours)	13% of time (hours)	3% of time (hours)	73% of patients (without midazolam), 84% of time (hours) (maintenance phase)		
Harper, *et al*., 1991 [25]	RCT of sedative drugs	37	Alfentanil low, moderate and high doses -- results reported together		4 patients had >10% of time at sedation level 6	3 patients had >10% of time at sedation level 1		Ramsay (assessed hourly)	2-5
Manley, *et al*., 1997 [46]	RCT (and economic study) of sedative drugs	26	Morphine + midazolam	56.8% of time			43.2% of time	North Staffordshire ICU (modification of Ramsay/Addenbrooke's scores)	3-4
			Alfentanil + propofol	57.8% of time			42.2% of time		
Millane, 1992 [21]	RCT of sedative drugs	24	Isoflurane for 24 hours followed by propofol	3.4%				Ramsay plus subjective nurse assessment	2-3 (plus subjective nurse assessment)
			Propofol for 24 hours followed by isoflurane	3.6%					
Muellejans, *et al*., 2004 [41]	RCT of sedative drugs	152	Remifentanil	11.7% of time (hours)			88.3% of time (hours)	SAS	4
			Fentanyl	10.7% of time (hours)			89.3% of time (hours)		
Muellejans, *et al*., 2006 [47]	RCT of sedative drugs	80	Remifentanil -- propofol	41% of time	28% of time	13% of time	59% of time	3 level sedation score specific to study	Level 2
			Midazolam -- fentanyl	30% of time	19% of time	11% of time	70% of time		
Chamorro, *et al*., 1996 [45]	RCT of sedative drugs	98	Propofol	332 assessments -- 3% (after first hour)			332 assessments -- 76.5% effective, 20.5% acceptable	Study-specific (modified Glasgow coma scale). Patients monitored at 1 and 6 hours and then every 12 hours.	4 = effective, 3 = acceptable less than 3 = ineffective
			Midazolam	355 assessments -- 7.6%			355 assessments -- 66.2% effective, 26.2% acceptable		
Barr, *et al*., 2001 [34]	RCT of sedative drugs	24	Lorazepam	51% of time	47% of time		49% of time	Modified Ramsay	3-4 (5-6 = over-sedation)
			Midazolam	31% of time	22% of time		69% of time		
Finfer, *et al*., 1999 [33]	RCT of sedative drugs	40	Diazepam (intermittent)	9 (64.3%) of 14 patients; 15.0% of time (hours)	2.8% of time (hours)	21.1% of time (hours)	5 (35.7%) of 14 patients; 85.0% of time (hours)	Modified Ramsay	1-4
			Midazolam (continuous)	6 (35.3%) of 17 patients; 40.8% of time (hours)	14.8% of time (hours)	0% of time (hours)	11 (64.7%) of 17 patients; 59.2% of time (hours)		
Richman, *et al*., 2006 [37]	RCT of sedative drugs	30	Midazolam	Mean 9.1 hours/day (SD 4.9)				Modified Ramsay	Individual to each patient
			Midazolam and fentanyl	Mean 4.2 hours/day (SD 2.4)					
Karabinis, *et al*., 2004 [39]	RCT of sedative drugs	161	Remifentanil	4.4% of time			95.6% of time (median)	SAS	1-3
			Fentanyl	1.9% of time			98.1% of time (median)		
			Morphine	1.0% of time			99.0% of time (median)		
Pandharipande, *et al*., 2007 [48], Pandharipande, *et al*., 2006 [59]	RCT of sedative drugs	106	Dexmedetomidine	20% of patients according to nurse goals; 33% according to physician goals	15% of patients		80% of patients within 1 point of nurse goal; 67% within 1 point of physician goal	RASS, confusion-assessment method for the ICU (CAM-ICU)	Individual to each patient
			Lorazepam	33% of patients according to nurse goals; 45% according to physician goals	33% of patients		67% within 1 point of nurse goal; 55% within 1 point of physician goal		
Swart, *et al*., 1999 [50]	RCT of sedative drugs	64	Lorazepam	13% of time			87.0% of time (SD 10.5)	Addenbrooke's Hospital's ICU sedation scale	Individual to each patient
			Midazolam	34% of time			66.2% of time (SD 23.1)		
Carson, *et al*., 2006 [22]	RCT of sedative drugs	132	Intermittent lorazepam	42.8% (ventilator hours)	37.9% (ventilator hours)	15.1% (ventilator hours)		Ramsay	2-3
			Continuous propofol	49.9% (ventilator hours)	38.6% (ventilator hours)	11.5% (ventilator hours)			
Anis, *et al*., 2002 [31], Hall, *et al*., 2001 [60]	RCT of sedative drugs	156	Propofol	39.8% of time	12.0% of time	11.2% of time	60.2% of time	Ramsay	Individual to each patient
			Midazolam	56.0% of time	18.4% of time	8.1% of time	44.0% of time		
Park, *et al*., 2007 [49]	RCT of sedative drugs	134 (111 analysed)	Analgesia-based sedation	50% of time			50% of time on SIMV (median)	Assessor judgement	Adequate judged as awake or easily rousable
			Hypnotic-based sedation	81% of time			19% of time on SIMV (median)		
Cigada, *et al*., 2005 [32]	Observational study of sedative drugs	42	Propofol or midazolam with enteral hydroxyzine with or without supplemental lorazepam. IV drugs were tapered after 48 hours.	36.9% of assessments as judged by Ramsay score; 17% by nurse assessment	421 (24.6%) of 1,711 assessments (Ramsay score) 42 (7.3%) of 577 assessments (nurse judgement)	211 (12.3%) of 1,711 assessments (Ramsay score) 56 (9.8%) of 577 assessments (nurse judgement)	1,079 (63.1%) of 1,711 assessments (Ramsay score) 479 (83%) of 577 assessments (nurse judgement)	Ramsay score plus nurse assessment	Adequate sedation defined as the achievement of the planned Ramsay score or nurse judgement as adequate
Barrientos-Vega, *et al*., 2001 [29]	Observational study of sedative drugs	51	2% propofol (compared with historical cohort on 1% propofol -- not reported here)	8 (15.6%) of 51 patients judged therapeutic failure on 2% propofol (inadequate level of sedation)				Ramsay score	4-5
MacLaren, *et al*., 2007 [42]	Observational study of sedative drugs	40	Dexmedetomidine as adjunct to lorazepam/midazolam/propofol	35% of patients with dexmedetomidine; 52% without	12 (30%) patients with dexmedetomidine; 9 (23%) without	4 (10%) patients with dexmedetomidine; 12 (30%) without	65% of patients with dexmedetomidine; 48% without	SAS	3-4
Shehabi, *et al*., 2004 [24]	Observational study of sedative drugs	20	Dexmedetomidine with supplemental midazolam if required	455 (33%) of 1,381 assessments	97 (7%) of 1,381 assessments were Ramsay level 6	137 (10%) of 1,381 assessments were Ramsay level 1	926 (67%) of 1,381	Ramsay	2-4
Sackey, *et al*., 2004 [51]	RCT of sedation devices	40	Isoflurane using AnaConDa	46% of time; nursing staff estimate 11% of time	44% of time	2% of time	54% of time; nursing staff estimate 89% of time	Bloomsbury scale	- 1 to +1
			IV midazolam	41%; nursing staff estimate 13% of time	37% of time	4% of time	59% of time; nursing staff estimate 87% of time		
Walsh, *et al*., 2008 [52]	Observational study of sedation devices	30	All sedated patients		137 (32.9%) of 416 assessments (Ramsay score 5-6)	5 (1.2%) of 416 assessments (Ramsay score 1)		Entropy Module/Modified Ramsay scale	None stated. Refers to guidelines suggesting 2-3 is adequate and heavy/over-sedated is 5-6.
Hernández-Gancedo, *et al*., 2006 [28]	Observational study of sedation scales	50			44% (66 cases) -- Ramsay level 6		25% (38 cases)	Ramsay, Observer's Assessment of Alertness and Sedation	Ramsay 3-4
Roustan, *et al*., 2005 [27]	Observational study of sedation scales	40	All sedated patients -- treated with midazolam and morphine		93 (61.6%) of 151 records	19 (12.6%) of 151 records		Ramsay, Comfort score, EEG	Ramsay 3-4
McMurray, *et al*., 2004 [38]	Observational study of sedation scales	122	Propofol-containing regimens	15.6% of time	Mean 5.0% of time (SD 12.7)	Mean 10.6% of time (SD 14.5)	Mean 84.4% of time (SD 18.0)	Modified Ramsay	Individual to each patient
Detriche, *et al*., 1999 [53]	Before-after study of introduction of sedation protocol	55	Before			20 (30%) of 67 assessment days		Brussels sedation scale	3-4
			After protocol introduction			9 (12%) of 77 assessment days			
Costa, *et al*., 1994 [54]	RCT of controlled and empirical sedation	40	Controlled	17% of time			83% of time	Ramsay, and Glasgow coma scale modified by Cook and Palma	
			Empirical	65% of time			35% of time		
MacLaren, *et al*., 2000 [35]	Before-after comparison of sedation protocol	158	Before			22.4% (experience of anxiety or pain)		Modified Ramsay	4
			After			11.0% (*P *< 0.001)			
Tallgren, *et al*., 2006 [3]	Before-after comparison of sedation protocol	53	Before reinforcement			Median Ramsay level was 4 during the day and 5 at night, in contrast to the study's stated aim of Ramsay level 2-3 during the day and 3-4 at night			Ramsay
			After reinforcement			Median Ramsay level was 4 during the day and 5 at night, in contrast to the study's stated aim of Ramsay level 2-3 during the day and 3-4 at night			
Samuelson, *et al*., 2007 [61], Samuelson, *et al*., 2006 [62]	Observational study	250			50% of patients had MAAS 0-2 (although 2 was target for study, 0-1 could be viewed as over-sedated)	0%	39% of patients achieved MAAS 3 in ventilated period	MAAS	Stated 2-3 but results reported for patients achieving 3

EEG, electroencephalogram; ICU, intensive care unit; IV, intravenous; MAAS, Motor Activity Assessment Scale; RASS, Richmond Agitation-Sedation Scale; RCT, randomised controlled trial; SAS, Riker Sedation-Agitation Scale; SD, standard deviation; SIMV, synchronised intermittent mandatory ventilation.

In addition to the variation in scales used to assess sedation, there was variation in the recommended range of optimal sedation levels stated. Sedation requirements obviously differ among patients; nevertheless, the variation in recommended ranges in included studies indicates some uncertainty in what constitutes optimal sedation. Of the studies using the Ramsay scale, recommended ranges were 2 to 3 (recommended in two studies [21,22]), 2 to 4 (two studies [23,24]), 2 to 5 (two studies [25,26]), 3 to 4 (two studies [27,28]) and 4 to 5 (one study [29]), while three studies did not recommend specific levels but recommended that levels be optimised for each individual patient [30-32]. This variation was reflected in the other scales used; for studies recommending a modified Ramsay scale, recommended ranges were 1 to 4 [33], 3 to 4 [34], 4 [35], and 5 to 6 (the last range being specifically for seriously injured patients [36]) or targets optimised for each patient [37,38]. The stated SAS (Riker Sedation-Agitation Scale) target level was 1 to 3 [39], 4 [40,41], or 3 to 4 [42]. Due to the number of studies recommending that optimal sedation state be determined individually for each patient, there was no comparison possible for other scales.

### Incidence of sub-optimal sedation

Table 1 lists the study design, sedation assessment scale or tool used, and incidence of sub-optimal sedation reported by studies. As stated above, we used individual study definitions of optimal and sub-optimal sedation because of the fact that optimal sedation levels are likely to vary by study setting.

The three observational studies that investigated the epidemiology of sedation were considered to be the most relevant to the study question as their specific aim was to investigate clinical sedation practice rather than practice within the confines of a trial, where more frequent monitoring and the Hawthorne effect could contribute to improving standards.

A survey of practice across 44 ICUs in France also found a high incidence of deep sedation, in 41% to 57% of readings over a 6-day period [43]. This study highlighted the risks of prolonged deep sedation, which, however, was not specifically defined as over-sedation. Results from these three studies indicate that 30% to 60% of sedation assessments indicate 'deep' or 'over' sedation, although precise description of the prevalence is confounded by imprecise definition or health care worker perceptions. These studies clearly indicate an excess of over-sedation compared with under-sedation.

Martin and colleagues [30] conducted a postal survey of 220 ICUs in Germany. This study found that 42.6% of patients sedated between 24 and 72 hours and 39.5% of patients sedated over 72 hours were over-sedated; the incidence of under-sedation was much lower (<6%).

In the US-based study of Weinert and colleagues [44], the aim was to compare subjective and objective ratings of sedation. Subjects provided 12,414 sedation assessments and were judged by nurses to be sub-optimally sedated in 17% of assessments, over-sedated in 2.6%, and under-sedated in 13.9%. Critically, however, patients were unrousable or minimally rousable just under one third of the time, indicating a high incidence of deep sedation. This finding illustrates the importance of the perception of the health care worker or assessor or both in describing the prevalence of sub-optimal sedation.

**Figure 3:**
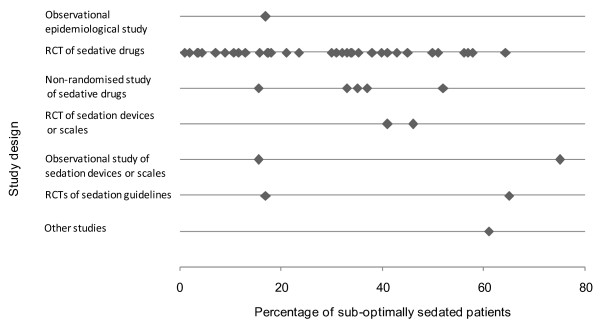
Incidence of sub-optimal sedation across included studies. The plot shows the percentage of measurements, patients, or time in which patients were sub-optimally sedated according to each included study's definition of optimal sedation and measurements reported. Studies are grouped by study design. Where more than one group was reported by a study (for example, a comparison of two different treatment arms), separate points are shown for each group. RCT, randomised controlled trial.

The remaining included studies comprised studies of sedative drugs [21-26,29,31-34,36,37,39-42,45-50], studies investigating different sedation devices or scales [27,28,38,51,52], and studies looking at the introduction of a sedation guideline or protocol [3,35,53,54]. Studies varied by design and aim, by sedatives used, by scales and definitions of sub-optimal sedation used, and by the way incidence was reported (as a proportion of measurements, patients, or time). While these studies did not necessarily have the incidence of sub-optimal sedation as their primary focus, the data in such studies were considered to be of interest to the inclusive scope of this review. Although studies of sedative drugs or of the introduction of guidelines or protocols may not give an accurate estimate of the incidence of sub-optimal sedation within routine clinical practice, they nevertheless show that it does occur and can give an impression of the extent to which it may be a problem, even in settings that could be reasonably expected to be more controlled than in routine practice. The incidence of sub-optimal sedation reported in these studies is summarised in Figure 3 (separated by study and treatment arm where relevant). The reported incidence varied from 1% [39] to 75% [28], with the majority reporting an incidence of over 20%. The incidence of over- and under-sedation was similarly variable, and figures of between 2.8% and 44% for over-sedation [28,33,51] and between 2% and 31% for under-sedation [23,51] were reported. A further study [2] that looked at the introduction of a sedation guideline did not record the incidence of sub-optimal sedation but recorded the median Ramsay scale values. These were 4 during the day and 5 at night, in contrast to the study's stated aim of Ramsay levels of 2 to 3 during the day and 3 to 4 at night; this study again noted a possible tendency toward over-sedation of patients. Importantly, there was no change in this tendency before and after reinforcement of the guideline, suggesting that this was insufficient to improve sedation practice [3].

## Discussion

Our systematic review identified few studies that specifically described the epidemiology of sedation during ICU care. Description of the incidence of sub-optimal sedation and over- and under-sedation was difficult due to variation in the use of these terms within individual studies. Overall, available data suggest a high incidence of over-sedation in ICUs, potentially present at 40% to 60% of assessments. A lower reported incidence of sub-optimal sedation across most studies suggests that health care workers consider deep levels of sedation appropriate for many patients.

The quality of published studies was low. There was wide variation in the method used to assess sedation state, the frequency of measurement, and the stated response to evaluations. In addition, the completeness of data in relation to entire ICU populations was usually not stated, introducing the potential for selection bias. Only three cohort studies were found. The importance of selection or inclusion bias was lowest with this study design. All of these indicated a substantial incidence of sub-optimal sedation, with over-sedation being more common (33% to 57%). Notably, one study reported that nurse assessment of sedation found a low incidence of over-sedation, which appeared at odds with the fact that in one third of measurements patients were unrousable or minimally rousable. A difference in perceptions of what constitutes optimal sedation between different health care worker groups and between individual health care workers is also likely to affect the reported incidence of sub-optimal sedation. This finding emphasises the importance of using sedation-assessment methods that have high validity and low inter-rater variability. Many of the scales in current use have not been subjected to formal evaluation. The RASS has been shown to have good construct validity and high levels of consistency among health care workers [55] but was used infrequently in the published literature.

When non-cohort studies were considered, a wide range of incidence of sub-optimal sedation levels was reported (from 1% to 75%). These studies were randomised or non-randomised trials of the efficacy of sedative drugs [21-26,29,31-34,36,37,39-42,45-50], or were studies that evaluated sedation devices or scales [27,28,38,51,52] to monitor sedation levels, or looked at the introduction of sedation guidelines [3,35,53,54]. Overall, the chance of selection and investigator bias, and of study effects, in these studies was high. Drug trials in particular were considered less relevant to our study question as practice within a clinical trial may differ from standard care and is more likely to be controlled. Despite these factors, the majority of studies found an incidence of sub-optimal sedation, using study-specific definitions, of greater than 20%. These studies confirm the findings of the observational cohort studies and suggest high levels of sub-optimal sedation during routine care.

Improving sedation management through sedation protocols and interventions such as daily interruption of sedation is an increasing focus of quality improvement initiatives in critical care in some health care systems [1,56,57]. Sedation protocols and scales are increasingly, though not universally, used. A review of German hospitals by Martin and colleagues [6] showed increases in the use of sedation protocols (from 21% of hospitals to 46%) and in the use of sedation scales (from 8% to 51%) over the period of 2002 to 2006. In Finland, Tallgren and colleagues [3] reported that reinforcing a sedation guideline increased the percentage of expected Ramsay scale recordings made, but only to 71% of expected recordings that were actually made, indicating that formal sedation assessments were still not carried out as regularly as they should have been. These studies suggest that, despite recent evidence supporting the avoidance of over-sedation [57,58], the use of systematic approaches to measure sedation state and optimise sedation for individual patients is not universal. The high prevalence of sub-optimal sedation and high incidence of over-sedation in published studies indicate potential for significant quality improvement in this aspect of care. This is likely to translate into substantial patient benefit.

There are several limitations to our review. As with any systematic review, studies may have been missed; this review was confined to English-language publications and therefore may be biased toward the US and UK in focus. Despite the inclusion of conference searching, there may be relevant grey literature that we did not search. As previously discussed, many of the included studies did not investigate the quality of sedation practice as their primary aim, limiting the relevance of the information provided.

## Conclusions

Our review indicates the poor quality of epidemiological data concerning current sedation practice and the incidence of sub-optimal sedation. A key issue is the standardisation of methods of assessment and definitions of optimal sedation. Despite this, available data suggest that many patients in ICUs are considered sub-optimally sedated and, specifically, that the incidence of over-sedation remains high. The strong associations between sedation practice, especially over-sedation, and adverse patient outcomes suggest that a more uniform approach to monitoring depth and quality of sedation will improve quality of care.

## Key messages

• The literature shows that, within the intensive care unit (ICU), a substantial proportion of patients experience inappropriate levels of sedation (that is, under- or over-sedation).

• There is a greater tendency toward over-sedation in particular.

• There is a lack of consensus in the literature as to what constitutes optimal sedation practice within the ICU, with little standardisation of either assessment methods or definitions.

• Improvements in the definition and measurement of optimal sedation may have a positive impact on patient outcomes.

## Abbreviations

BIS: bispectral index monitor; ICU: intensive care unit; RASS: Richmond Agitation-Sedation Scale; RCT: randomised controlled trial.

## Competing interests

The systematic review reported in this publication was funded by GE Healthcare (Chalfont St. Giles, UK). DLJ is an employee of GE Healthcare. CWP and KFC are employed by Heron Evidence Development Ltd (Luton, UK), which was commissioned to undertake research by GE Healthcare. The article processing charge was funded by GE Healthcare. GE Healthcare has developed a device for monitoring consciousness levels in sedated patients TW is collaborating with GE Healthcare in developing a sedation monitoring device. His institution has received research funding from GE for collaborative research, but TW has not gained personally and has not received any direct payment from GE Healthcare. TW holds no shares in GE Healthcare and is not an employee of the company.

## Authors' contributions

DLJ conceived the study and helped with manuscript revisions. CWP designed and performed searches, extracted data and wrote the manuscript draft. KFC researched and wrote the treatment guidelines section and assisted with data extraction for the main systematic review. TW provided expert clinical input and worked on manuscript revisions. All authors read and approved the final manuscript.

## Authors' information

TW is a professor of anaesthetics and critical care at Edinburgh University. DLJ is head of health economics, EMEA (Europe, the Middle East and Africa), at GE Healthcare. CWP is a consultant at Heron Evidence Development Ltd, a health outcomes research consultancy. KFC is a health outcomes analyst at Heron Evidence Development Ltd.
